# Effect of tuberculosis screening and retention interventions on early antiretroviral therapy mortality in Botswana: a stepped-wedge cluster randomized trial

**DOI:** 10.1186/s12916-019-1489-0

**Published:** 2020-02-11

**Authors:** Andrew F. Auld, Tefera Agizew, Anikie Mathoma, Rosanna Boyd, Anand Date, Sherri L. Pals, Christopher Serumola, Unami Mathebula, Heather Alexander, Tedd V. Ellerbrock, Goabaone Rankgoane-Pono, Pontsho Pono, James C. Shepherd, Katherine Fielding, Alison D. Grant, Alyssa Finlay

**Affiliations:** 1 Division of Global HIV & TB, Centers for Disease Control and Prevention, Atlanta, USA; 2 Center for Global Health, U.S. Centers for Disease Control and Prevention (CDC), Lilongwe, Malawi; 3 Division of TB Elimination, Centers for Disease Control and Prevention, Gaborone, Botswana; 4 Ministry of Health, Gaborone, Botswana; 5 Yale University School of Medicine, New Haven, CT USA; 6 TB Centre, London Sch. of Hygiene & Tropical Med, London, UK; 7 School of Public Health, University of the Witwatersrand, Johannesburg, South Africa; 8 Africa Health Research Institute, School of Nursing and Public Heath, University of KwaZulu-Natal, Durban, South Africa

**Keywords:** Tuberculosis, Xpert MTB/RIF, Intensified tuberculosis case finding, Mortality

## Abstract

**Background:**

Undiagnosed tuberculosis (TB) remains the most common cause of HIV-related mortality. Xpert MTB/RIF (Xpert) is being rolled out globally to improve TB diagnostic capacity. However, previous Xpert impact trials have reported that health system weaknesses blunted impact of this improved diagnostic tool. During phased Xpert rollout in Botswana, we evaluated the impact of a package of interventions comprising (1) additional support for intensified TB case finding (ICF), (2) active tracing for patients missing clinic appointments to support retention, and (3) Xpert replacing sputum-smear microscopy, on early (6-month) antiretroviral therapy (ART) mortality.

**Methods:**

At 22 clinics, ART enrollees > 12 years old were eligible for inclusion in three phases: a retrospective standard of care (SOC), prospective enhanced care (EC), and prospective EC plus Xpert (EC+X) phase. EC and EC+X phases were implemented as a stepped-wedge trial. Participants in the EC phase received SOC plus components 1 (strengthened ICF) and 2 (active tracing) of the intervention package, and participants in the EC+X phase received SOC plus all three intervention package components. Primary and secondary objectives were to compare all-cause 6-month ART mortality between SOC and EC+X and between EC and EC+X phases, respectively. We used adjusted analyses, appropriate for study design, to control for baseline differences in individual-level factors and intra-facility correlation.

**Results:**

We enrolled 14,963 eligible patients: 8980 in SOC, 1768 in EC, and 4215 in EC+X phases. Median age of ART enrollees was 35 and 64% were female. Median CD4 cell count was lower in SOC than subsequent phases (184/μL in SOC, 246/μL in EC, and 241/μL in EC+X). By 6 months of ART, 461 (5.3%) of SOC, 54 (3.2%) of EC, and 121 (3.0%) of EC+X enrollees had died. Compared with SOC, 6-month mortality was lower in the EC+X phase (adjusted hazard ratio, 0.77; 95% confidence interval, 0.61–0.97, *p* = 0.029). Compared with EC enrollees, 6-month mortality was similar among EC+X enrollees.

**Conclusions:**

Interventions to strengthen ICF and retention were associated with lower early ART mortality. This new evidence highlights the need to strengthen ICF and retention in many similar settings. Similar to other trials, no additional mortality benefit of replacing sputum-smear microscopy with Xpert was observed.

**Trial registration:**

Retrospectively registered: ClinicalTrials.gov (NCT02538952)

## Background

In resource-limited settings, tuberculosis (TB) remains the most common cause of death among people living with HIV (PLHIV), including those starting antiretroviral therapy (ART), and is commonly undiagnosed at the time of death [1, 2]. Death from undiagnosed TB or TB diagnosed late is a key reason early (6-month) ART mortality rates remain significantly higher in sub-Saharan Africa (SSA) than resource-rich settings [2–4]. All data point towards a critical need to improve TB case finding among PLHIV starting ART.

In 2011, following World Health Organization (WHO) endorsement of Xpert MTB/RIF® (Xpert) as the first-line TB diagnostic test for symptomatic PLHIV [5], the Botswana Ministry of Health (MOH) and partners initiated planning for a phased national Xpert rollout [6]. Review of available program data for new HIV care enrollees showed that many components of the intensified TB case finding (ICF) cascade, especially compliance with the WHO-recommended 4-symptom TB screening rule, and early retention in HIV care, should be strengthened in order for Xpert to have maximum benefit [7]. Weaknesses in the health system that have resulted in poor completion of the TB diagnostic and treatment cascade and sub-optimal retention in HIV care, have been cited as important reasons for lack of observed Xpert impact on PLHIV mortality in similar settings [8, 9]. Therefore, Botswana used the Xpert rollout as an opportunity to strengthen ICF and retention in early HIV care through rollout of a package of services [6]. The intervention package has three components: (1) additional support for ICF, (2) intensified tracing for patients missing clinic appointments to return them to care, and (3) Xpert replacing sputum-smear microscopy.

No trial has yet evaluated impact of Xpert combined with strengthened health systems on mortality [8–10]. We evaluated impact of the Xpert, ICF, and retention package versus standard of care on early ART patient mortality.

## Methods

### Study design

We conducted a multi-center, stepped-wedge cluster randomized trial (CRT) with a retrospective baseline component called the Xpert Package Rollout Evaluation using a Stepped-wedge design (XPRES) trial. A stepped-wedge rather than parallel group design was chosen because the Xpert, ICF, and retention package was expected to be beneficial for patients and the trial was part of a national rollout [6].

### Participants

**Fig. 1:**
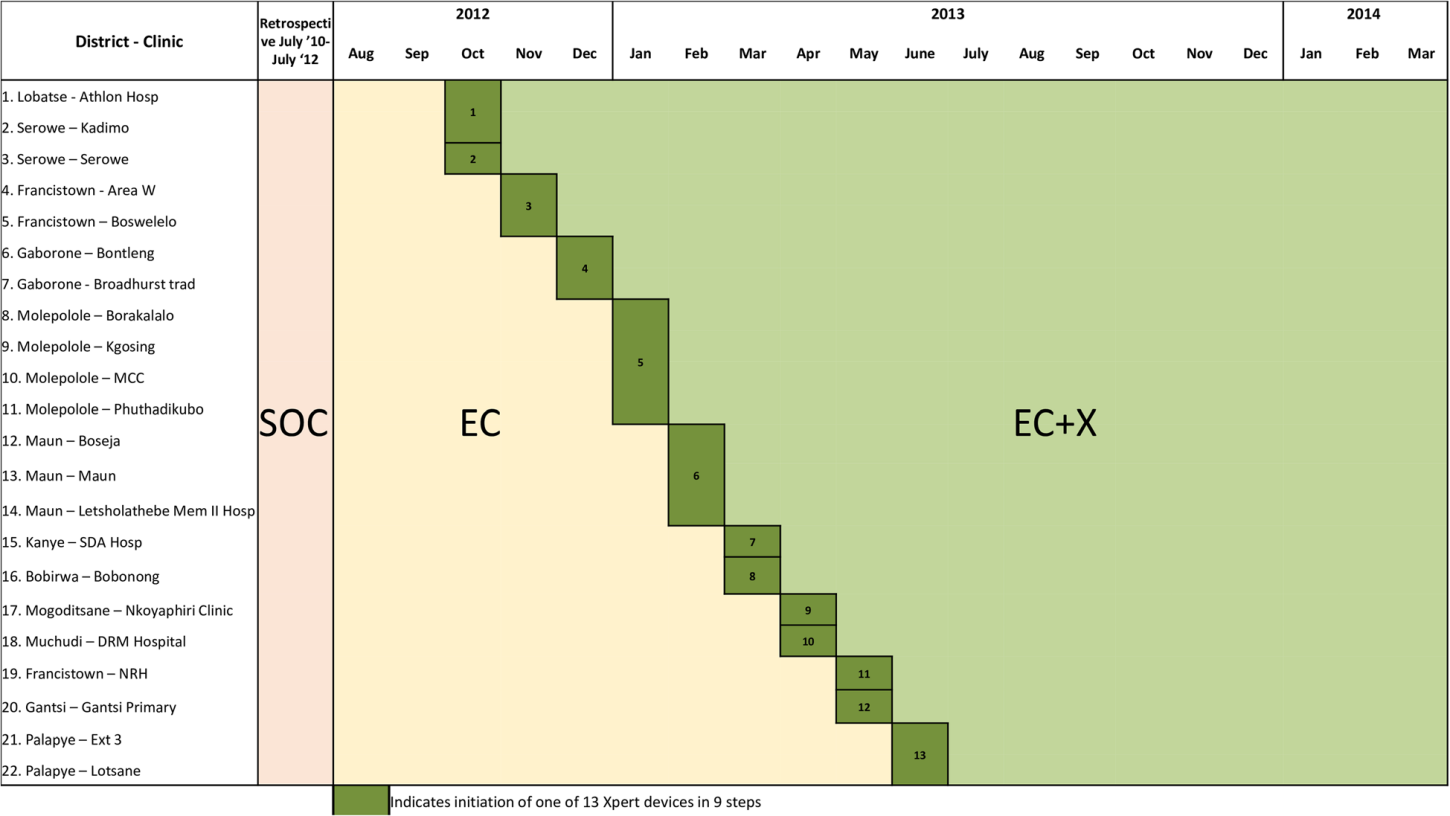
Study design for the Xpert Package Rollout Evaluation using a Stepped-wedge design (XPRES). Abbreviations: SOC, standard of care phase; EC, enhanced care phase; EC+X, enhanced care plus Xpert phase

A cluster was defined as an HIV care and treatment clinic. Twenty-two clusters, located at five district hospitals and 17 primary healthcare facilities, were purposively selected to (1) be representative of HIV treatment clinics in Botswana and (2) have new ART initiation rates sufficient to meet sample size requirements (see Additional file 1, providing text on clinic selection criteria). At these 22 clusters, individual patients were eligible for study enrollment if they were new HIV clinic attendees, regardless of TB treatment status, and not prisoners at the time of the first HIV clinic visit. The study aimed to enroll or offer enrollment to all eligible HIV clinic attendees in three consecutive phases: (1) a retrospective standard of care (SOC) phase, (2) a prospective enhanced care (EC) phase, and (3) a prospective EC plus Xpert (EC+X) phase (Fig. 1). For this pre-defined protocol analysis, only those study enrollees who newly started ART at or after study enrollment and were ≥ 12 years old at ART initiation were included [6].

### Randomization and masking

The selected 22 clusters received TB diagnostic services from 13 laboratories (Fig. 1). Because some of the study clinics used the same TB diagnostic laboratory, full Xpert, ICF, and retention package activation was planned to be simultaneous for these clinic consortiums (Fig. 1). After obtaining ethical approvals and agreement to participate in the study from MOH at a central level and MOH management at the selected facilities, the study statistician randomly selected one of the rollout permutations [6].

### Procedures

At the 22 clusters, per Botswana national guidelines during the time period of the study (July 2010 through June 2015), all study participants in all phases were eligible for ART initiation if they had a CD4 count ≤ 350 cells/μL, were diagnosed as having WHO stage III/IV, or were pregnant or breastfeeding [11]. All study participants received clinical care and follow-up appointments according to MOH guidelines (see Additional file 2, a table summarizing standard clinical care follow-up).

#### Standard of care phase

Enrollment in the retrospective SOC phase was through chart abstraction of eligible adult patients who started ART between July 2010 and the end of July 2012 (Fig. 1) [6]. The SOC phase enrollees received HIV care according to national guidelines, limited ICF, infrequent active tracing due to resource limitations, and sputum-smear microscopy for presumptive TB patients.

#### Intervention phases EC and EC+X

Prospective EC enrollment started in August 2012 and was complete by January 2013. Prospective EC+X enrollment occurred from October 2012 through March 2014 according to the stepped-wedge design (Fig. 1). EC phase participants received SOC supplemented by two components of the Xpert, ICF, and retention package (i.e., additional support for ICF and intensified tracing) combined with sputum-smear microscopy. EC+X phase participants received SOC supplemented by all three components of the Xpert, ICF, and retention package (i.e., additional support for ICF, intensified tracing, and Xpert in place of sputum-smear microscopy). All interventions were activated at the cluster-level for the benefit of all clients receiving care at the clinic. EC and EC+X participants were followed for 12 months, or until the end of TB treatment, whichever was later. The final follow-up visits for EC+X enrollees were in June 2015.

#### Interventions

The ICF and active tracing interventions were strengthened through four key mechanisms: (1) additional human resources (study nurses) to support implementation, (2) additional training for clinic and laboratory personnel, (3) use of checklists and job aids to standardize implementation, and (4) regular supervisory visits to track adherence to ICF and tracing checklists.

#### ICF intervention

Implementation of the WHO 4-symptom TB screening rule (i.e., screening for cough of any duration, fever, loss of weight, and night sweats) [12] was recommended for all enrollees at each clinic visit in the SOC, EC, and EC+X phases, but implementation was strengthened in the EC and EC+X phases. In all phases, clients were considered symptomatic if they screened positive for one or more of the four TB symptoms. In all phases, at least two same-day, on-the-spot (spot) sputum samples were recommended for collection from symptomatic clients. As part of strengthened ICF in the EC and EC+X phases, a previously published job-aid was used by study nurses to inform the patient how to collect quality sputum samples [6]. Prior to the EC phase, laboratory personnel at the 13 laboratories serving the 22 clusters received refresher training on Ziehl-Neelsen staining for sputum-smear microscopy, and prior to the EC+X phase, laboratory personnel were trained for Xpert implementation. In all phases, sputum test results were returned to the clinics, with clinicians responsible for informing the patients. In the SOC phase, the patient was informed of a TB diagnosis at the next scheduled clinic appointment. In the EC and EC+X phases, study nurses were trained to work with laboratories to ensure the turnaround time from sample collection to result return to the clinic was ≤ 4 days for sputum-smear microscopy and ≤ 2 days for Xpert testing. In the EC and EC+X phases, nurses were trained to inform patients of positive TB diagnoses the same day via phone, or if unreachable by phone, by active tracing to the household. Indicators monitoring implementation of the ICF cascade were collected and used to inform supervision visits (see Additional file 3, a table summarizing the indicators) [7].

#### Active tracing intervention

Per national guidelines, clients ≥ 1 day late for an HIV clinic appointment should be traced through phone and home visit starting the day after the missed visit. However, program reports showed this tracing was infrequently implemented in the SOC phase due to lack of human and financial resources. Implementation of the active tracing policy was strengthened in the EC and EC+X cohorts. In the EC and EC+X phases, a patient locator form was used to document telephone numbers and home addresses for intensified tracing activities to support retention. Up to five telephone calls and two home visits, facilitated by checklists, were used in attempts to return clients, who had missed clinic appointments, to care. The key HIV care retention indicator used for monitoring purposes was the rate of loss to follow-up (LTFU) per 100 person-years (see Additional file 3, a table summarizing the indicators). LTFU was defined as being > 60 days late for a scheduled appointment, per Botswana guidelines.

### Objectives and outcomes

The study had two primary objectives. The primary objective reported here is the non-randomized comparison of all-cause 6-month ART mortality among adult ART enrollees (≥12 years old) between the SOC and EC+X phases [6]. The second primary objective, which aimed to compare diagnostic sensitivity of the new Xpert-based TB diagnostic algorithm with that of the sputum-smear-microcopy-based algorithm, will be reported separately according to diagnostic accuracy study reporting guidelines.

Secondary objectives reported in this paper include (1) the comparison of 12-month ART mortality between SOC and EC+X phases and (2), within the randomized stepped-wedge trial, the comparison of all-cause, adult, 6-month ART mortality between the EC and EC+X phases.

We implemented intensive efforts to ascertain true mortality outcomes among participants. Deaths and date of death were either passively reported to the clinic by friends or relatives of the deceased participant, or actively ascertained if the client had missed an appointment or was considered LTFU [13]. Initial efforts to ascertain outcomes of clients who missed an appointment or were LTFU included phone outreach to the client or contact and home visits. For participants in the SOC phase, these efforts started after data entry was complete which was always > 12 months after ART initiation. In the EC and EC+X phases, this outreach started immediately after the missed appointment, in an attempt to return the client to care. For all clients unreachable by phone or home visit who met the LTFU definition, vital status was ascertained through national Death Registry review. By law, since 1969, all deaths need to be registered in the Death Registry, which is maintained by the Civil and National Registration Office.

### Sample size

As described previously [6], to obtain conservative sample size estimates, we used the approach of Moulton et al., suitable for stepped-wedge trial designs, to estimate required sample sizes to meet the primary study objective comparing 6-month ART mortality rates between SOC and EC+X phases [14]. Funding limitations restricted the number of clinics that could be included in the study to 22. A between-cluster coefficient of variation of 0.2 was used based on review of the literature of similar stepped-wedge trials [14]. Monthly HIV clinic (cluster) size was derived from reported program ART enrollment rates in the SOC phase and varied between clinics (average, 23 ART enrollees/month; range, 8–46/month). Prior to study start, available data from Botswana suggested that all-cause, adult, 6-month ART mortality rates were about 15 deaths per 100 person-years [3, 15]. To provide > 80% power to detect a ≥ 40% reduction in all-cause 6-month ART mortality between the two groups, assuming SOC mortality was ≥ 10/100 person-years, a 24-month SOC phase enrollment period (*N* = 12,144) and an 18 month EC+X phase enrollment period (*N* = 6348) were chosen.

### Statistical analysis

For the primary outcome analysis, time at risk for ART enrollees started on the day of ART initiation and ended at 6 months of follow-up after ART initiation, or at the time of death, LTFU, or transfer out if these events were before 6 months of ART follow-up. Crude and multivariable Cox proportional hazards regression models, with a random effect for clinic, were used to assess the effect of intervention status (SOC vs EC+X) on time to death [6]. Per a pre-specified analysis plan, age at ART initiation, sex, pregnancy status, and baseline CD4 count were a priori covariates to be included in the multivariable model. Hemoglobin at ART initiation [16], ART regimen [17], and weight at ART initiation [16] were included in the multivariable model because of their importance as predictors of mortality in this and other analyses.

Pre-specified secondary analyses were conducted to (1) compare 12-month ART mortality between SOC and EC+X phases and (2) compare 6-month ART mortality rates between cohorts EC and EC+X [6]. For the latter, we used analytic methods described by Moulton et al., fitting Cox proportional hazards models to the data with the underlying time frame being time since August 2012 (initiation month for the stepped-wedge component of the trial), fixed effect for intervention arm (Xpert device activation), and a random effect for clinic [14]. The proportionality assumption was checked using visual methods and the Grambsch and Therneau test.

Per the pre-specified analysis plan, plausible interactions between the intervention effect and other covariates, including CD4 count at ART initiation, were examined by comparing models with and without interactions using the likelihood ratio test. Per the pre-specified analysis plan, the primary time-to-event analytic approaches comparing SOC versus EC+X and EC versus EC+X mortality rates assigned follow-up time to the phase in which the participant started ART because the interventions were expected to have maximum impact around the time of ART initiation. However, two pre-specified sensitivity analyses of this approach were planned. The first sensitivity analysis censors follow-up time for ART enrollees at the time of cross-over between phases, while the second assigns follow-up time to contemporary intervention phases when cross-over occurs, through use of a time-dependent covariate [18]. In addition, per a third pre-specified sensitivity analysis, an inverse probability weighting approach was used to account for non-enrollment in the EC and EC+X phases of the study. Separate adjusted logistic regression models for hospital versus clinic enrollees were used to predict the probability of being enrolled in the study. Patients consenting to enrollment were up-weighted by the inverse of the calculated enrollment probability. An adjusted logistic regression approach was used to estimate inverse probability weights to lower the likelihood of bias given the possibility of non-random enrollment in the EC and EC+X phases [19]. All analyses were conducted using STATA 14 or 16 (StataCorp, 2009, Stata Statistical Software, Release 14 and 16, College Station, TX). XPRES is registered at ClinicalTrials.gov (trial registration no. NCT02538952).

## Results

### Enrollment

**Fig. 2:**
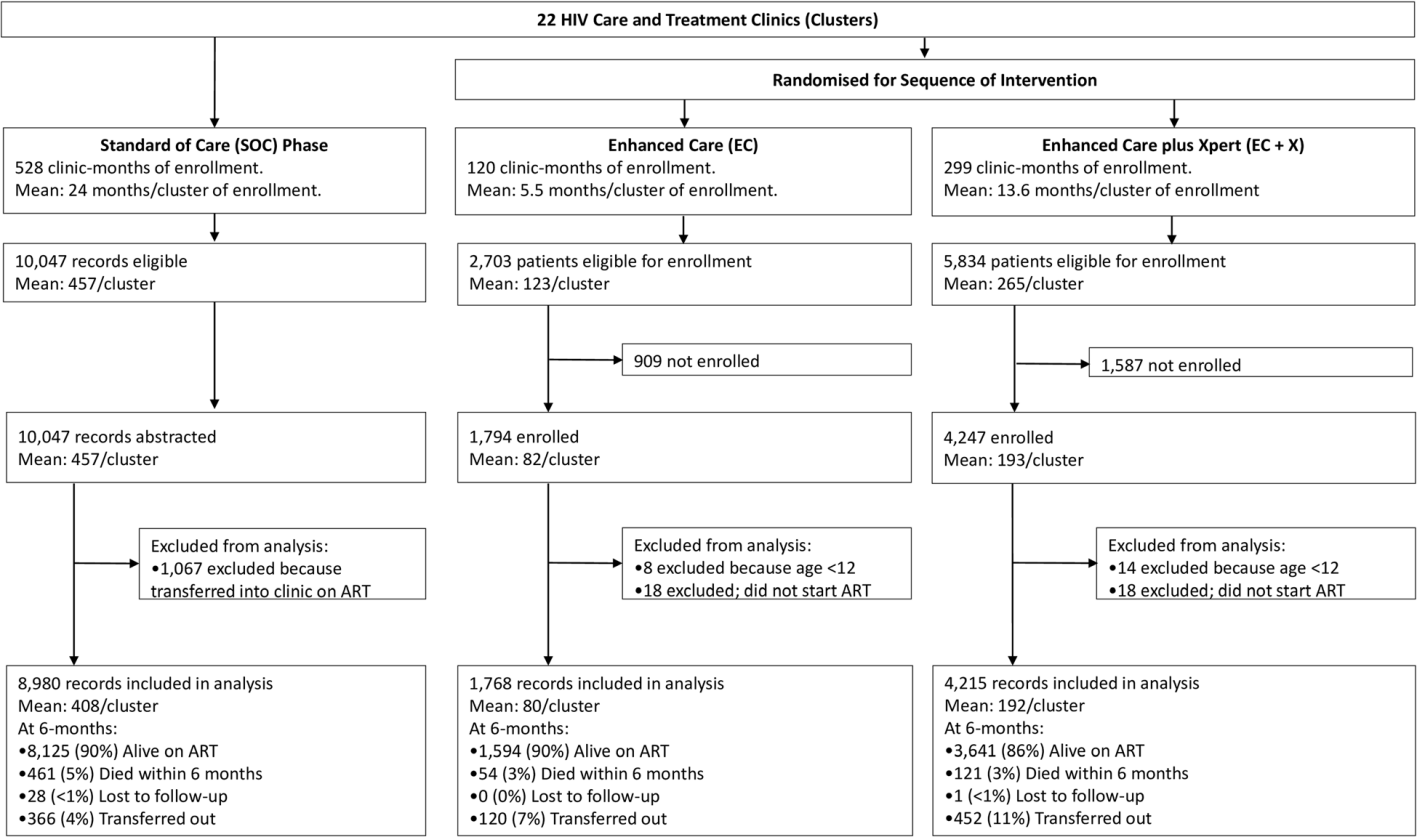
Trial profile

Across the 22 study clinics, there were 528 months of enrollment in the SOC phase (mean 24/clinic), 120 months in the EC phase (mean 5.5/clinic), and 299 months of enrollment in the EC+X phase (mean 13.6 months/clinic) (Fig. 2). All 10,047 eligible patients for the SOC phase were enrolled. Among the 2703 and 5834 patients eligible for the EC and EC+X phases, respectively, 1794 (66%) and 4247 (73%) consented to enrollment. The main reason eligible clients were not enrolled prospectively is that they left the clinic before they could be offered enrollment. The demographic and clinical characteristics of clients consenting to enrollment were very similar to the characteristics of clients not enrolled (see Additional file 4, a table comparing characteristics of those enrolled versus not enrolled). We excluded from this analysis patients who transferred into the clinic on ART (*n* = 1067), were < 12 years old at ART initiation (*n* = 22), or did not start ART during follow-up (*n* = 36) (Fig. 2). In total, 8980, 1768, and 4215 patients were included in the SOC, EC, and EC+X phases for analysis, respectively.

### Baseline characteristics

Among all study enrollees included in the analysis, median age was 35 (interquartile range (IQR) 29–42) at ART initiation and the percentage female was 64% and these characteristics were similar between phases (Table 1). Among female enrollees, the percentage who were pregnant at the time of ART initiation was lower in the SOC phase (16%) than EC (23%) and EC+X (32%) phases. Among all enrollees, median weight (58.4 kg) and median hemoglobin (11.7 g/dL) were similar between phases. However, median CD4 count at ART initiation was lower in the SOC phase (184 cells/μL) than in the EC (246 cells/μL) and EC+X (241 cells/μL) phases. In addition, the percentage of enrollees with mild or moderate anemia per WHO criteria was higher in the SOC phase (56%) than EC (48%) and EC+X phases (46%). Tenofovir (combined with lamivudine or emtricitabine and efavirenz or nevirapine) was less commonly prescribed as first-line ART in the SOC (78%) compared with the EC (93%) and EC+X (96%) phases.

**Table 1:** Demographic and clinical characteristics of XPRES participants at antiretroviral therapy initiation

	SOC		EC		EC+X	
	(*N* = 8980)		(*N* = 1768)		(*N* = 4215)	
	*n*	%/median (IQR)	*n*	%/median (IQR)	*n*	%/median (IQR)
Age (years)^a^						
*n*, median, (IQR)	8969	35 (30–43)	1768	34 (29–42)	4215	34 (29–41)
Gender						
Female	5624	63%	1194	68%	2797	66%
If female, pregnant?						
Yes	927	16%	271	23%	903	32%
Weight (kg)^b^						
Median (IQR)	8351	57.9 (50.5–66.6)	1765	58.6 (51.3–67.8)	4209	59.4 (52.5–68.7)
Weight (kg)						
< 45 kg	871	10%	160	9%	318	8%
45–60 kg	3971	48%	817	46%	1910	45%
> 60 kg	3509	42%	788	45%	1981	47%
Baseline CD4 (cells/μL)^c^						
Median (IQR)	8675	184 (100–241)	1765	246 (148–310)	4180	241 (132–321)
Baseline CD4 (cells/μL)						
< 50	1061	12%	132	7%	370	9%
50 to < 100	1109	13%	161	9%	371	9%
100 to < 200	2660	31%	366	21%	928	22%
200 to < 350	3456	40%	947	54%	1928	46%
350 to < 500	246	3%	93	5%	334	8%
≥ 500	143	2%	66	4%	249	6%
Baseline hemoglobin (g/dL)^d^						
Median (IQR)	7869	11.5 (10.0–13.0)	1678	11.9 (10.4–13.3)	3911	12.0 (10.6–13.3)
Hemoglobin category^e^						
Severe anemia	426	5%	68	4%	109	3%
Mild/moderate anemia	4399	56%	805	48%	1810	46%
No anemia	3044	39%	805	48%	1992	51%
TB treatment at ART initiation						
Yes	423	5%	85	5%	251	6%
Regimen^f^						
TDF/XTC/EFV or NVP	6998	78%	1615	93%	4000	96%
AZT/3TC/EFV or NVP	1045	12%	94	5%	107	3%
D4T/3TC/EFV or NVP	151	2%	2	0%	4	0%
Other	784	9%	26	1%	54	1%

Abbreviations: *SOC* standard of care phase, *EC* enhanced care phase, *EC+X* enhanced care plus Xpert phase, *IQR* interquartile range, *TDF* tenofovir, *XTC* either lamivudine or emtricitabine, *EFV* efavirenz, *NVP* nevirapine, *ddI* didanosine, *ABC* abacavir, *LPV/r* lopinavir/ritonavir, *AZT* zidovudine, *3TC* lamivudine, *D4T* stavudine

^a^11 ART enrollees in the SOC cohort had unknown age but were documented to be adult in the ART chart

^b^629 (7%), 2 (0.2%), and 6 (0.1%) had missing weights at ART initiation in the SOC, EC, and EC+X phases, respectively

^c^305 (3%), 3 (0.2%), and 35 (0.8%) had missing CD4 in the SOC, EC, and EC+X phases, respectively. For each enrollee, the CD4 count taken closest to the date of ART initiation in the 12 months before ART start was used

^d^1111 (12%), 90 (5%), and 304 (7.2%) had missing hemoglobin in the SOC, EC, and EC+X phases, respectively. For each enrollee, the hemoglobin taken closest to the date of ART initiation in the 12 months before ART start was used

^e^Anemia severity was classified according to World Health Organization criteria as follows: no anemia, hemoglobin level of ≥ 13.0 g/dL for men, ≥ 12.0 g/dL for non-pregnant females, and ≥ 11.0 g/dL for pregnant females; mild/moderate anemia, 8.0 to < 13.0 g/dL for men, 8.0 to < 12.0 g/dL for non-pregnant women, and 7.0 to < 11.0 g/dL for pregnant women; and severe anemia, < 8.0 g/dL for males and non-pregnant females and < 7.0 g/dL for pregnant women

^f^2 (0%), 31 (2%), and 50 (1%) had missing ART regimen in the SOC, EC, and EC+X phases, respectively

### Primary outcome: 6-month ART mortality in SOC versus EC+X

**Fig. 3:**
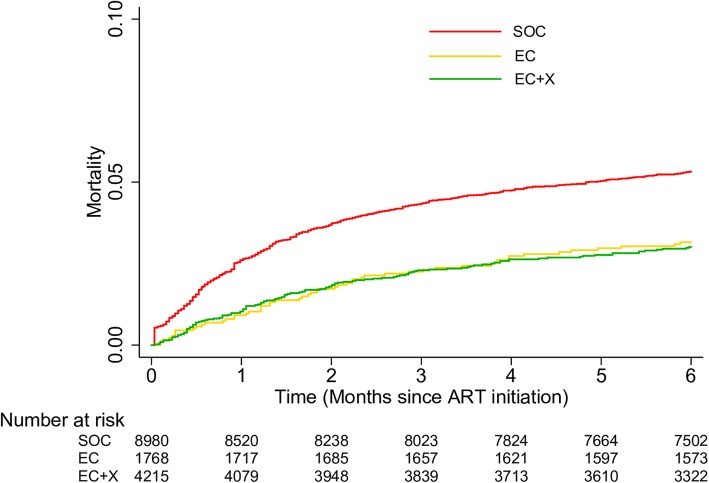
Kaplan-Meier curves showing cumulative 6-month mortality among ART enrollees in SOC, EC, and EC+X phases

By 6 months after ART initiation, 461 (5.3%) of enrollees in the SOC phase had died compared with 121 (3.0%) of enrollees in the EC+X phase. Six-month ART mortality rates were 11.4 deaths per 100 person-years in the SOC phase versus 6.3 deaths per 100 person-years in the EC+X phase (Table 2). Compared with the SOC phase, 6-month mortality was lower in the EC+X phase in unadjusted analysis (hazard ratio (HR) 0.58, 95% CI 0.48–0.71, *p* < 0.001) (Fig. 3, Table 2). After controlling for potential confounders, including age, sex, pregnancy status, weight, CD4 count, hemoglobin, and ART regimen, 6-month mortality remained lower in the EC+X phase compared with the SOC phase (adjusted HR, 0.77, 95% CI 0.61–0.97, *p* = 0.029).

**Table 2:** Primary and secondary study outcomes—comparison of mortality rates between study phases

	ART enrollees	Deaths (*n*)^a^	Rate/100PY^b^	Crude HR^c^	(95% CI)	*p*	AHR^cd^	(95% CI)	*p*
Primary outcome: 6-month ART mortality in SOC versus EC+X phase									
SOC	8980	461	11.4	1.00	–	–	1.00	–	–
EC+X	4215	121	6.3	0.58	(0.48–0.71)	< 0.001	0.77	(0.61–0.97)	0.029
Secondary outcomes: 12-month ART mortality in SOC versus EC+X phase									
SOC	8980	551	7.3	1.00	–	–	1.00	–	–
EC+X	4215	137	4.6	0.58	(0.48–0.70)	< 0.001	0.76	(0.61–0.95)	0.014
6-month ART mortality in EC versus EC+X phase^e^									
EC	1768	54	6.5	1.00			1.00		
EC+X	4215	121	6.3	1.07	(0.62–1.84)	0.800	1.13	(0.63–2.03)	0.690

Abbreviations: *SOC* standard of care phase, *EC* enhanced care phase, *EC+X* enhanced care plus Xpert phase, *PY* person-years, *HR* hazard ratio, *AHR* adjusted hazard ratio, *CI* confidence interval, *XPRES* Xpert Package Rollout Evaluation using a Stepped-Wedge design

^a^Represents deaths observed among all ART enrollees by the time point specified

^b^Represents unadjusted 6- and 12-month ART mortality rates among all ART enrollees in each phase of the study. For mortality rates among ART enrollees included in the adjusted analyses, see Additional file 6

^c^All Cox proportional hazards regression models included a random effect for clinic

^d^Adjusted for the following characteristics at ART initiation: age, sex, pregnancy status, weight, CD4 count, hemoglobin, and ART regimen. Adjusted analysis comparing SOC versus EC+X mortality rates included 7184 SOC enrollees with 350 deaths within 6 months and 424 deaths within 12 months, and 3861 EC+X enrollees with 93 deaths within 6 months and 108 deaths within 12 months

^e^Analysis restricted to randomized stepped-wedge portion of the trial, fitting a Cox proportional hazards regression model to the data with the underlying time frame beginning August 2012 (the start of EC enrollment), and including a fixed effect for monthly changes in mortality rates during the first 6 months of ART. Adjusted analysis comparing EC versus EC+X mortality rates included 1653 EC enrollees with 43 deaths within 6 months and 3861 EC+X enrollees with 93 deaths within 6 months

Intervention effect size was similar across CD4 strata (see Additional file 5, a figure showing cumulative mortality incidence stratified by CD4 count at ART initiation). In addition, effect size was robust to sensitivity analyses that censored follow-up time at the time of transition between phases or assigned follow-up time to contemporary intervention phases using a time-dependent covariate (see Additional file 6, a table showing these sensitivity analyses). Effect size was robust to sensitivity analysis using an inverse probability weighting approach to account for non-enrollment in EC and EC+X phases (see Additional file 7, a table showing these sensitivity analyses).

### Secondary outcomes: 12-month ART mortality in SOC versus EC+X

By 12 months after ART initiation, 551 (6.5%) of SOC versus 137 (3.7%) of EC+X phase enrollees had died. Twelve-month mortality rates were 7.3/100 person-years in the SOC versus 4.6/100 person-years in the EC+X phase. Compared with the SOC phase, 12-month mortality was lower in the EC+X phase in both unadjusted (HR 0.58, 95% CI 0.48–0.70, *p* < 0.001) and adjusted (AHR 0.76, 95% CI 0.61–0.95, *p* = 0.014) analyses (Table 2). Intervention effect size was robust to sensitivity analyses (see Additional files 6 and 7, tables showing sensitivity analyses).

### Secondary outcomes: 6-month ART mortality in EC versus EC+X

By 6 months of ART follow-up among ART enrollees in the EC phase, 54 (3.2%) of enrollees had died. Six-month mortality rates were similar between the EC (6.5/100 person-years) and EC+X phases (6.3/100 person-years) in both unadjusted and adjusted pre-specified analyses (AHR 1.13, 95% CI, 0.63–2.03), where all follow-up time was assigned to the phase in which the patient started ART (Table 2). In sensitivity analyses comparing EC vs. EC+X 6-month mortality rates, the AHR was 0.90 (95% CI 0.42–1.95) when EC enrollee follow-up time was censored at the time of EC+X cross-cover, and 0.79 (95% CI 0.41–1.50) when EC enrollee follow-up time in the EC+X phase was assigned to the EC+X phase using a time-dependent variable (see Additional file 6, a table showing sensitivity analyses).

### TB screening and diagnosis

**Fig. 4:**
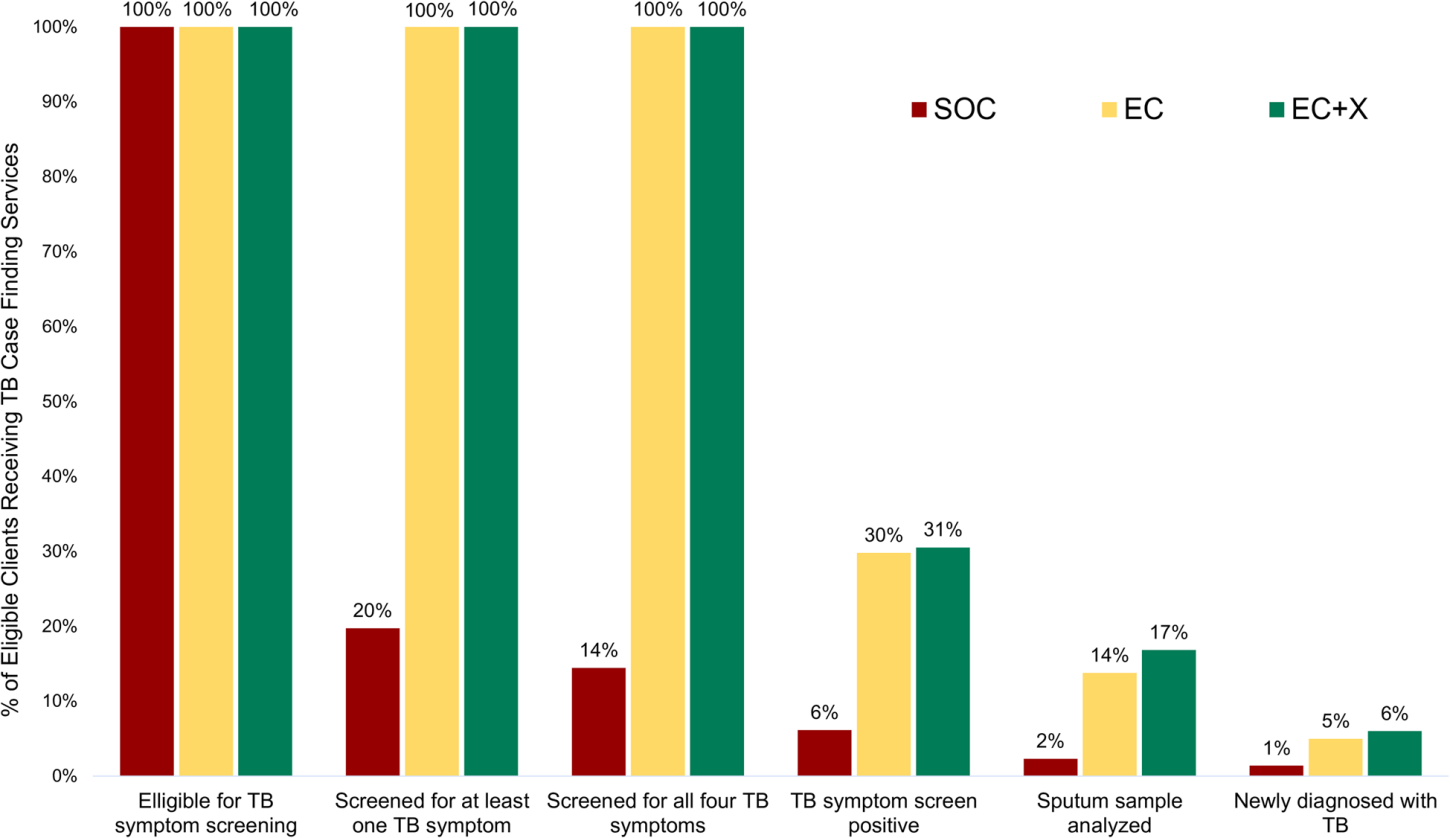
Intensified TB case finding (ICF) cascade among ART enrollees in SOC, EC, and EC+X phases. Abbreviations: SOC, standard of care phase, EC, enhanced care phase, EC+X, enhanced care plus Xpert phase

Among SOC, EC, and EC+X phase enrollees respectively, 359 (4%), 44 (2%), and 122 (3%) were diagnosed with TB and had started TB treatment prior to arrival at the HIV treatment clinic. Therefore, in the SOC, EC, and EC+X phases, 8621, 1724, and 4093 patients were eligible for TB symptom screening before ART initiation. Among these patients eligible for TB symptom screening before ART initiation in the SOC, EC, and EC+X phases, 1700 (20%), 1724 (100%), and 4093 (100%) were screened for at least one TB symptom and 1243 (14%), 1724 (100%), and 4093 (100%) were screened for all four TB symptoms, respectively (Fig. 4). Within the SOC phase, ART enrollees were more likely to be screened for at least one TB symptom if they had lower weight and lower CD4 count at ART initiation (see Additional file 8, a table showing predictors of being screened for TB in the SOC cohort).

Among SOC, EC, and EC+X enrollees eligible for screening, 525 (6%), 514 (30%), and 1249 (31%) screened positive for at least one TB symptom and 199 (2%), 237 (14%), and 688 (17%) provided a sputum sample for TB diagnosis (Fig. 4). Ultimately, 129 (1%), 86 (5%), and 244 (6%) enrollees in the SOC, EC, and EC+X phases were newly diagnosed with TB and started TB treatment before ART initiation or during the first 6 months of ART. The number of pulmonary TB diagnoses in the SOC (*n* = 123), EC (*n* = 68), and EC+X (*n* = 198) phases that were confirmed microbiologically was 22 (18%), 35 (51%), and 129 (65%), respectively (Table 3).

**Table 3:** Methods of new TB diagnosis immediately before ART and in the first 6 months of ART in the SOC, EC, and EC+X phases of XPRES

	SOC phase		EC phase		EC+X phase	
	*n*	%	*n*	%	*n*	%
Pulmonary TB						
Microbiologically confirmed pulmonary TB (smear microscopy in SOC and EC, Xpert during EC+X)	22	18	23	34	113	57
Microbiologically confirmed pulmonary TB through culture^a^ (missing or negative smear and Xpert)	0	0	12	18	16	8
Clinical diagnosis of pulmonary TB with negative sputum test (negative smear, Xpert, or culture documented)	6^b^	5	6^d^	9	17^f^	9
Clinical diagnosis of pulmonary TB with no documented sputum test result	95^c^	77	27^e^	40	52^g^	26
Sub-total pulmonary TB	123	100	68	100	198	100
All TB						
Pulmonary TB total	123	95	68	79	198	81
Extra-pulmonary TB total	6	5	18	21	46	19
Total	129	100	86	100	244	100

Abbreviations: *SOC* standard of care, *EC* enhanced care, *EC+X* enhanced care plus Xpert, *TB* tuberculosis, *XPRES* Xpert Package Rollout Evaluation using a Stepped-wedge design

^a^To meet other study objectives related to estimation of diagnostic accuracy of the smear microscopy-based and Xpert-based TB diagnostic algorithms, one spot sputum and the morning sputum were sent to the National TB Reference Laboratory (NTRL) for liquid culture in mycobacteria growth indicator tubes (MGIT). The liquid culture results were also returned to the clinics, although average turnaround times exceeding 49 days were expected per existing standard of care

^b^5 (83%) of 6 had documentation that x-ray findings were suggestive of pulmonary TB

^c^13 (14%) of 95 had documentation that x-ray findings were suggestive of pulmonary TB

^d^3 (50%) of 6 had documentation that x-ray findings were suggestive of pulmonary TB

^e^16 (59%) of 27 had documentation that x-ray findings were suggestive of pulmonary TB

^f^8 (47%) of 17 had documentation that x-ray findings were suggestive of pulmonary TB

^g^20 (38%) of 52 had documentation that x-ray findings were suggestive of pulmonary TB

### Early ART LTFU

By 6 months after ART initiation, cumulative LTFU incidence, uncorrected by subsequent mortality ascertainment efforts, in the SOC, EC, and EC+X phases, was 4%, 1%, and 1%, respectively (see Additional file 9, a table summarizing these cumulative LTFU incidence percentages). Compared with 6-month LTFU rates in the SOC phase (8.3/100 person-years), rates of 6-month LTFU were lower in the EC (1.2/100 person-years) and EC+X (1.6/100 person-years) phases in both unadjusted and adjusted analyses (see Additional file 10, a table comparing LTFU rates between SOC, EC, and EC+X phases).

## Discussion

In Botswana, compared with SOC, interventions to strengthen WHO-recommended TB symptom screening and ICF algorithms combined with active tracing to support retention were associated with increased TB case finding and lower early ART mortality. No additional mortality benefit of replacing sputum-smear microscopy with Xpert was observed.

Although implementation of the WHO-recommended 4-symptom TB screening rule as the first step in ICF algorithms among PLHIV starting ART has been recommended since 2011 along with TB-HIV care continuum retention interventions including active tracing [20], no study has yet reported on the potential impact on mortality of strengthening systems to implement these guidelines [7]. Although the observed reduction in all-cause mortality between SOC and subsequent EC and EC+X phases represents a pre- versus post-comparison, rather than a randomized comparison, and is therefore at risk of residual confounding, the study has a number of strengths that suggest ICF and retention interventions did independently contribute to observed mortality impact. Firstly, the reduction in all-cause mortality remained statistically significant after adjusting for key covariates. Secondly, the improvements in TB screening, TB case finding, and uncorrected LTFU rates between SOC and subsequent EC and EC+X phases were large, providing credence that these interventions were a driver behind observed mortality reductions. Thirdly, very high ascertainment of the primary early ART mortality outcome improves ability to interpret observed mortality changes. Fourthly, the intervention effect size and statistical significance were robust to several sensitivity analyses. Therefore, these findings represent important additional evidence in support of current WHO ICF and retention guidelines, and support continued or additional investment from donors to strengthen health systems to implement these guidelines for all HIV clinic enrollees [9].

Although it was widely anticipated that introduction of the new more sensitive TB diagnostic test (Xpert) in place of sputum-smear microscopy would independently reduce mortality among PLHIV, this study and six of the seven previously reported Xpert impact trials have not observed any independent impact of Xpert versus sputum-smear microscopy on mortality [8, 21]. In the one trial that did observe Xpert impact on mortality, the mortality benefit was restricted to clients with advanced HIV disease (WHO stage III/IV) [21]. Furthermore, program data have clearly shown that leaks in the ICF cascade before a TB diagnostic test is implemented, especially failure to implement the WHO-recommended 4-symptom TB screen, may be largely responsible for unacceptably high rates of mortality due to undiagnosed TB among PLHIV engaged in care in sub-Saharan Africa [22, 23].

Per WHO guidelines, screening for the four TB symptoms (i.e., current cough, weight loss, night sweats, or fever) should occur at every clinical care encounter for PLHIV as the initial step in ICF to improve detection and treatment of HIV-associated TB [20]. The recommendation is based on a high sensitivity of the 4-symptom screening rule (89.4%) in detecting culture-positive pulmonary TB disease among ART-naïve PLHIV [24]. However, low compliance in implementing the 4-symptom TB screen at or prior to ART initiation has been consistently observed in many high burden TB-HIV countries in sub-Saharan Africa, including South Africa (59%) [23], Mozambique (61%) [25], Kenya (4%) [26], and Cote d’Ivoire (36%) [22]. Similarly, in XPRES, failure to implement TB screening before ART was the most “leaky” part of the ICF cascade in the SOC phase, with only 30% screened before ART. Improving the coverage of TB symptom screening from 30% in the SOC to 100% in the EC and EC+X phases was the main driver behind improved TB case detection from 1% in SOC to 5–6% in EC and EC+X phases and therefore appears to have been a key driver behind the declines in early ART mortality between SOC and subsequent EC and EC+X phases.

Reasons for low compliance with TB screening protocols in the SOC phase are not well understood, but could have related to high patient load making healthcare workers more likely to omit key steps in care algorithms, inadequate training and knowledge of the guidelines, or deficiencies in monitoring and evaluation [27]. In the SOC phase, having more advanced disease at ART initiation (i.e., having a lower weight and CD4 count) was associated with higher odds of being screened for TB, suggesting that healthcare workers were triaging the clients to receive TB screening based on perception of disease stage. This finding might fit with a clinic experiencing high patient volume and HCW’s rushing through patient consultations in order to complete their clinical duties within available business hours. Our intervention of providing additional nurses to implement the TB screening, additional training, and additional supervision increased the percentage of ART enrollees screened for TB from 30% to 100%.

Notably, although the percentage of enrollees screening positive for ≥ 1 TB symptom who provided ≥ 1 sputum sample increased from 38% in the SOC phase to 46% and 55% in the EC and EC+X phases, respectively, collection of sputum samples remained a challenge even in the EC phases. This low compliance with sputum collection guidelines has been observed in multiple settings [23, 27], with potential reasons being patient hesitance to provide a sputum sample for stigma-related reasons, true inability to provide a sputum sample, and HCW-related reasons such as feeling overloaded, or lack of confidence in the laboratory sample transport and diagnostic system [23]. Further research and interventions to improve this component of the cascade are needed. In addition, this finding supports calls for improved sputum-independent diagnostic tests for TB.

A key reason that prior Xpert impact trials have generally not observed independent Xpert impact on mortality is that higher rates of empiric TB treatment among clients with TB symptoms but a negative sputum-smear microscopy result replaced any potential benefit of Xpert’s improved diagnostic sensitivity in detecting culture-positive TB [28, 29]. Similarly in our study, although Xpert implementation was the driver behind increased microbiological confirmation of TB diagnoses in the EC+X versus EC phase (65% vs. 51%), there was no significant difference in percentage of ART enrollees newly treated for TB (6% vs. 5%). However, as reported previously, Xpert was the driver behind reduced median time from sputum collection to TB treatment in the EC+X phase (6 days) versus the EC phase (22 days) [30]. Although no independent effect of Xpert on 6-month mortality was observed in our study, two features of the study suggest, similar to findings of a recent meta-analysis of Xpert impact trials [31], that we cannot confidently rule out the possibility of modest independent Xpert impact: (1) our study was not powered to detect a difference between EC and EC+X 6-month mortality and (2) the sensitivity analyses comparing EC vs. EC+X 6-month mortality rates generated AHRs of 0.90 (*p* = 0.793) and 0.79 (*p* = 0.472), which could possibly point to a modest Xpert impact our study was under-powered to detect.

In ART programs in resource-limited settings, observed LTFU from early ART is common, with an average of 20% LTFU by 12 months of follow-up [32, 33]. Mortality rates among LTFU ART patients are high [33]. The percentage of LTFU clients found to have died by the time of tracing ranges from 20 to 60% [13, 33]. In our study, 41% of patients LTFU in the first 6 months of ART in the SOC phase had died by 6 months of follow-up. Accumulating data show that among LTFU patients who have died by the time of tracing, mortality rates are highest shortly after the last clinic visit, the majority (> 90%) die from illness rather than other causes (e.g., trauma), and the majority had some opportunity for clinical intervention at the last visit [33]. In addition, six previous trials, which aimed to evaluate Xpert impact on patient-important outcomes, have reported that LTFU of patients with bacteriologically confirmed TB, either before or during TB treatment, almost certainly reduces the potential impact of improved TB case finding on mortality [8].

The reductions in LTFU achieved in EC and EC+X phases compared with the SOC phase are likely due to a combination of factors, including the strengthened tracing intervention, additional training and nurses, and possibly reduced incidence of missed visits due to inter-current illness from undiagnosed TB [34]. The intensified tracing intervention might be particularly helpful in maintaining a personalized partnership with clients struggling with adherence to clinic visit schedules for a variety of reasons to ensure minimal interruption in ART pill taking [34]. These data support the underlying principle that supportive services to retain patients in HIV care are an essential component of both the ICF and HIV treatment cascade.

The absence of an interaction between CD4 count at ART initiation and intervention package effect size suggests that ICF and retention interventions could be important for all new HIV clinic enrollees, not just those with advanced disease as defined by WHO (CD4 count < 200 copies/ml) [35]. Therefore, although median CD4 count at ART initiation is increasing in many countries, including Botswana [36], with most countries having adopted WHO universal HIV treatment guidelines, these data support current WHO recommendations that high-quality implementation of ICF and retention interventions remains important for HIV clinic enrollees.

This study has a number of strengths and limitations. Strengths include the large sample size, accurate ascertainment of the primary mortality outcome, and implementation in a real-world programmatic setting, which improves generalizability of findings. Limitations include the fact that the primary objective relies on an adjusted pre-post analysis that is subject to residual confounding, and that data from the SOC phase were collected retrospectively. In the SOC phase, TB screening or sputum sample collection may sometimes have been implemented but not documented. While retrospective data collection in the SOC phase increases the likelihood of missing covariate data, it also ensures that the type of care received by clients in the SOC phase truly represents the care provided prior to implementation of the EC and EC+X interventions. While EC and EC+X phases were of different duration, our study results show good compliance with ICF algorithm implementation and impressive active tracing impact on LTFU throughout EC and EC+X phases, indicating no discernable lag time needed for these interventions to reach maximum potential. In addition, good implementation of Xpert in the EC+X phase is evidenced by the increase in the percentage of TB cases that were microbiologically confirmed in EC+X versus EC phases, and in the shorter time from sputum collection to TB treatment in EC+X versus EC phases, with these results consistent with several prior Xpert impact trials [8]. Notably, while these data support effectiveness of the ICF and retention intervention in reducing early ART mortality, future economic evaluation would be needed to explore cost-effectiveness.

## Conclusions

In summary, a health system strengthening intervention to improve compliance with WHO-recommended TB symptom screening and ICF algorithms, combined with active tracing to support retention of HIV and HIV-TB co-infected patients in care through the early period of ART, was associated with significant reductions in early ART mortality and should be considered for scale-up. In addition, similar to most other trials of Xpert impact on mortality, replacing sputum-smear microscopy with Xpert was not associated with a mortality reduction.

## Supplementary information

**Additional file 1.** Text showing selection criteria for study clinics.

**Additional file 2.** Table of standard clinical follow-up of clients in SOC, EC, and EC+X phases (2010–2015).

**Additional file 3.** Table of indicators used to assess implementation of TB ICF and retention in the HIV care cascade.

**Additional file 4.** Table comparing demographic and clinical characteristics between prospective study enrollees in the EC and EC+X phases and eligible clients declining enrollment.

**Additional file 5.** Figure of cumulative 6-month ART mortality stratified by SOC, EC, and EC+X phases among (a) enrollees with CD4 < 200 cells/μL, (b) CD4 ≥ 200 cells/μL.

**Additional file 6.** Table of sensitivity analyses of primary and secondary study outcomes - comparison of mortality rates between study phases.

**Additional file 7.** Table of sensitivity analyses of primary and secondary study outcomes to account for non-response - comparison of mortality rates between study phases.

**Additional file 8.** Table showing predictors of being screened for at least one TB symptom in the standard of care phase of XPRES.

**Additional file 9.** Table comparing 6-month ART outcomes before versus after efforts to ascertain accurate primary mortality outcome status among clients LTFU by study phase.

**Additional file 10.** Table showing differences in rates of uncorrected loss to follow-up in the first 6 months of ART between SOC, EC, and EC+X phases.

## Data Availability

The datasets generated and/or analyzed during the current study are not publicly available due to an IRB decision which was made in the interest of ensuring patient confidentiality but are available from the corresponding author on reasonable request. Per IRB guidance, the datasets will be anonymized before sharing.
