# Supplementary material for: Effect of tuberculosis screening and retention interventions on early antiretroviral therapy mortality in Botswana: a stepped-wedge cluster randomized trial
Source: BMC Med. 2020 Feb 11;18:19. doi: 10.1186/s12916-019-1489-0 (PMC7011529; doi:10.1186/s12916-019-1489-0)
Supplement: Supplementary file 1 — Additional file 1. Text showing selection criteria for study clinics. [file 12916_2019_1489_MOESM1_ESM.docx]

**S1 - Text: Selection criteria for study clinics**

XPRES study clinics were purposively selected to be representative of ART clinics in Botswana, while also ensuring sample sizes could be reached. Clinic characteristics that were taken into account have been previously published and included:

- All 22 clinics had at least one year’s experience in providing ART services.
- 21 of 22 sites had ART enrollment rates >8 ART patients per month (mean 23/month; range 8-46/month) according to routine program data. These enrollment rates were anticipated to meet study sample size requirements. One site had an unknown enrollment rate at study initiation (Gantsi), but enrollment rates of eight ART enrollees/month were observed during study conduct.
- The study clinic with initially unknown ART enrolment rates (Gantsi) was selected because it was thought to have a high prevalence of MDR TB among HIV clinic enrollees and MOH believed these patients would benefit from early rollout of the Xpert device.
- Accessibility to either onsite or off-site TB laboratories was representative of HIV care and treatment centers in Botswana
- All sites were implementing the microscopy-based TB diagnostic algorithm prior to study initiation.
- All sites had the ability to perform testing, or to transport specimens for, hematology, serum chemistry, and CD4 count analysis, as is standard for ART sites in Botswana.
