# Supplementary material for: Effect of tuberculosis screening and retention interventions on early antiretroviral therapy mortality in Botswana: a stepped-wedge cluster randomized trial
Source: BMC Med. 2020 Feb 11;18:19. doi: 10.1186/s12916-019-1489-0 (PMC7011529; doi:10.1186/s12916-019-1489-0)
Supplement: Supplementary file 2 — Additional file 2. Table of standard clinical follow-up of clients in SOC, EC, and EC+X phases (2010–2015). [file 12916_2019_1489_MOESM2_ESM.docx]

**S2 - Table: Clinical follow-up of clients in SOC, EC, and EC+X phases (2010-2015)**

| **Pre-ART, CD4 >350** | **3 monthly** | Weight, CD4, TB screen |
| --- | --- | --- |
| **ART** | **ART start** | Weight, CD4, TB screen, ALT/AST if NVP-based regimen, Hb if AZT-based regimen, Hepatitis B screen, creatinine if TDF-based regimen |
|  | **2 weeks** | Weight, TB screen, ALT/AST if NVP-based regimen, Hb if AZT-based regimen |
|  | **1 month** | Weight, TB screen, ALT/AST if NVP-based regimen, Hb if AZT-based regimen |
|  | **3 months** | Weight, TB screen, ALT/AST if NVP-based regimen, Hb if AZT-based regimen, Viral load, creatinine if TDF-based regimen |
|  | **6months** | Weight, TB screen, ALT/AST^a^ if NVP-based regimen, Hb if AZT-based regimen, Viral load, CD4 |
|  | **Quarterly^b^** | Weight, TB screen, Viral load and CD4 6 monthly, creatinine if TDF-based regimen 6 monthly |

Abbreviations: CD4, CD4 cell count; TB, tuberculosis; ALT, alanine transaminase; AST, aspartate aminotransferase; NVP, nevirapine; AZT, zidovudine; TDF, tenofovir;

^a^Routine ALT/AST not required after 6 months but may be requested by the clinician depending on the clinical situation.

^b^For those patients started on PI-based regimens, baseline and 12-monthly glucose (random or fasting) and total cholesterol/triglycerides are recommended.
