# Supplementary material for: Effect of tuberculosis screening and retention interventions on early antiretroviral therapy mortality in Botswana: a stepped-wedge cluster randomized trial
Source: BMC Med. 2020 Feb 11;18:19. doi: 10.1186/s12916-019-1489-0 (PMC7011529; doi:10.1186/s12916-019-1489-0)
Supplement: Supplementary file 3 — Additional file 3. Table of indicators used to assess implementation of TB ICF and retention in the HIV care cascade. [file 12916_2019_1489_MOESM3_ESM.docx]

**S3 - Table: Indicators used to assess implementation of TB ICF and retention in the HIV care cascade**

**Indicators used to monitor the intensified TB case finding (ICF) cascade**

|  | **Indicator** | **Denominator and Numerator** |
| --- | --- | --- |
| 1 | % of ART enrollees screened for ≥1 WHO-recommended TB symptom before or on the day of ART initiation | **Denominator:**  The number of ART enrollees eligible for TB symptom screening (i.e., are not already diagnosed with TB).  **Numerator:**  The number of ART enrollees with documented screening for ≥1 of four WHO-recommended TB symptoms (cough, loss of weight, fever, night sweats) before or on the day of ART initiation |
| 2 | % of ART enrollees screened for all four WHO-recommended TB symptoms before or on the day of ART initiation | **Denominator:** Same as in #1  **Numerator:**  The number of ART enrollees with documented screening for all four WHO-recommended TB symptoms (cough, loss of weight, fever, night sweats) before or on the day of ART initiation |
| 3 | % of ART enrollees screening positive for ≥1 of four WHO-recommended TB symptoms before or on the day of ART initiation | **Denominator:** Same as in #1  **Numerator:**  The number of ART enrollees screening positive for ≥1 of four WHO-recommended TB symptoms before or on the day of ART initiation. |
| 4 | % of ART enrollees having a sputum sample analyzed at the lab to diagnose TB | **Denominator:** Same as in #1  **Numerator:**  The number of ART enrollees having a sputum sample sent for analysis on or before the date of ART initiation. |
| 5 | % of ART enrollees newly diagnosed with either extra-pulmonary or pulmonary TB | **Denominator:** Same as in #1  **Numerator:**  The number of ART enrollees newly diagnosed with TB before ART initiation or during the first 6 months of ART. |

**Indicators used to monitor loss to follow-up from ART**

|  | **Indicator** | **Denominator and Numerator** |
| --- | --- | --- |
| 1 | Rate of uncorrected^a^ LTFU from ART during the first 6 months of ART | **Denominator:**  Person-years of follow-up starting on the day of ART initiation and ending at the event of interest, which would be the date of last attended follow-up appointment for those LTFU, date of death, date of transfer out or departure from the study, or 6 months of follow-up if still alive and on ART at 6 months after ART initiation.  **Numerator:**  The number of clients meeting the definition of LTFU within the first 6 months of ART (i.e., >60 days late for the next scheduled appointment). |

^a^Mortality ascertainment efforts among all patients meeting the LTFU definition were implemented with subsequent correction of 6-month ART outcomes (see S7).
