# Supplementary material for: Effect of tuberculosis screening and retention interventions on early antiretroviral therapy mortality in Botswana: a stepped-wedge cluster randomized trial
Source: BMC Med. 2020 Feb 11;18:19. doi: 10.1186/s12916-019-1489-0 (PMC7011529; doi:10.1186/s12916-019-1489-0)
Supplement: Supplementary file 4 — Additional file 4. Table comparing demographic and clinical characteristics between prospective study enrollees in the EC and EC+X phases and eligible clients declining enrollment. [file 12916_2019_1489_MOESM4_ESM.docx]

**S4 – Table: Comparison of demographic and clinical characteristics between prospective study enrollees in the EC and EC+X phases and eligible clients declining enrollment**

|  | **EC** | | **EC+X** | | **Declined Enrollment in Prospective Cohorts** | |
| --- | --- | --- | --- | --- | --- | --- |
|  | **(N=1,768)** | | **(N=4,215)** | | **(N=2,439)^a^** | |
|  | **n** | **%/median (IQR)** | **n** | **%/median (IQR)** | **n** | **%/median (IQR)** |
| **Age** |  |  |  |  |  |  |
| n, Median, (IQR) | 1,768 | 34 (29-42) | 4,215 | 34 (29-41) | 2,439 | 34 (28-41) |
| Missing | 0 | 0% | 0 | 0% | 0 | 0% |
| **Gender** |  |  |  |  |  |  |
| Female | 1,194 | 68% | 2,797 | 66% | 1,650 | 68% |
| **If female, pregnant?** |  |  |  |  |  |  |
| Yes | 271 | 23% | 903 | 32% | 580 | 35% |
| **Weight (Kg)** |  |  |  |  |  |  |
| Median (IQR) | 1,765 | 58.6 (51.3-67.8) | 4,209 | 59.4 (52.5-68.7) | 2,246 | 60.3 (52.8-69.5) |
| Missing | 3 | 0.20% | 6 | 0.10% | 193 | 8% |
| **Weight (Kg)** |  |  |  |  |  |  |
| <45 kg | 160 | 9% | 318 | 8% | 177 | 8% |
| 45-60 kg | 817 | 46% | 1,910 | 45% | 931 | 41% |
| >60 kg | 788 | 45% | 1,981 | 47% | 1,138 | 51% |
| **Baseline CD4 (cells/µL)** |  |  |  |  |  |  |
| Median (IQR) | 1765 | 246 (148-310) | 4,180 | 241 (132-321) | 2,367 | 242 (139-322) |
| Missing | 3 | 0.20% | 35 | 0.80% | 72 | 3% |
| **Baseline CD4 (cells/µL)** |  |  |  |  |  |  |
| <50 | 132 | 7% | 370 | 9% | 195 | 8% |
| 50-<100 | 161 | 9% | 371 | 9% | 212 | 9% |
| 100-<200 | 366 | 21% | 928 | 22% | 517 | 22% |
| 200-<350 | 947 | 54% | 1,928 | 46% | 1,081 | 46% |
| 350-<500 | 93 | 5% | 334 | 8% | 206 | 9% |
| ≥500 | 66 | 4% | 249 | 6% | 156 | 7% |
| **Baseline Hemoglobin (g/dL)** |  |  |  |  |  |  |
| Median (IQR) | 1,678 | 11.9 (10.4-13.3) | 3,911 | 12.0 (10.6-13.3) | 2,169 | 11.9 (10.5-13.2) |
| Missing | 90 | 5% | 304 | 7.20% | 270 | 11% |
| **Hemoglobin category** |  |  |  |  |  |  |
| Severe anemia | 68 | 4% | 109 | 3% | 73 | 3% |
| Mild/moderate anemia | 805 | 48% | 1,810 | 46% | 1,017 | 47% |
| No anemia | 805 | 48% | 1,992 | 51% | 1,079 | 50% |

Abbreviations: SOC, standard of care phase; EC, enhanced care phase; EC+X, enhanced care plus Xpert phase; IQR, interquartile range;

^a^Note that a total of 2,496 patients declined to enroll. However, of these patients, 57 were <12 at the time of first presentation to the clinic and were therefore ineligible for this analysis and are not included in the column of clients declining enrolment to facilitate comparisons with EC and EC+X cohorts. Of all 2,439 clients >=12 at first presentation to the study clinic, 2,430 (99.6%) were documented to have started ART by the end of the prospective cohort enrollment period (March 31, 2014). The mean percentage of patients declining enrollment by clinic was 29% (range: 6%-51%).
