# Supplementary figures and images for: Effect of tuberculosis screening and retention interventions on early antiretroviral therapy mortality in Botswana: a stepped-wedge cluster randomized trial
Source: BMC Med. 2020 Feb 11;18:19. doi: 10.1186/s12916-019-1489-0 (PMC7011529; doi:10.1186/s12916-019-1489-0)

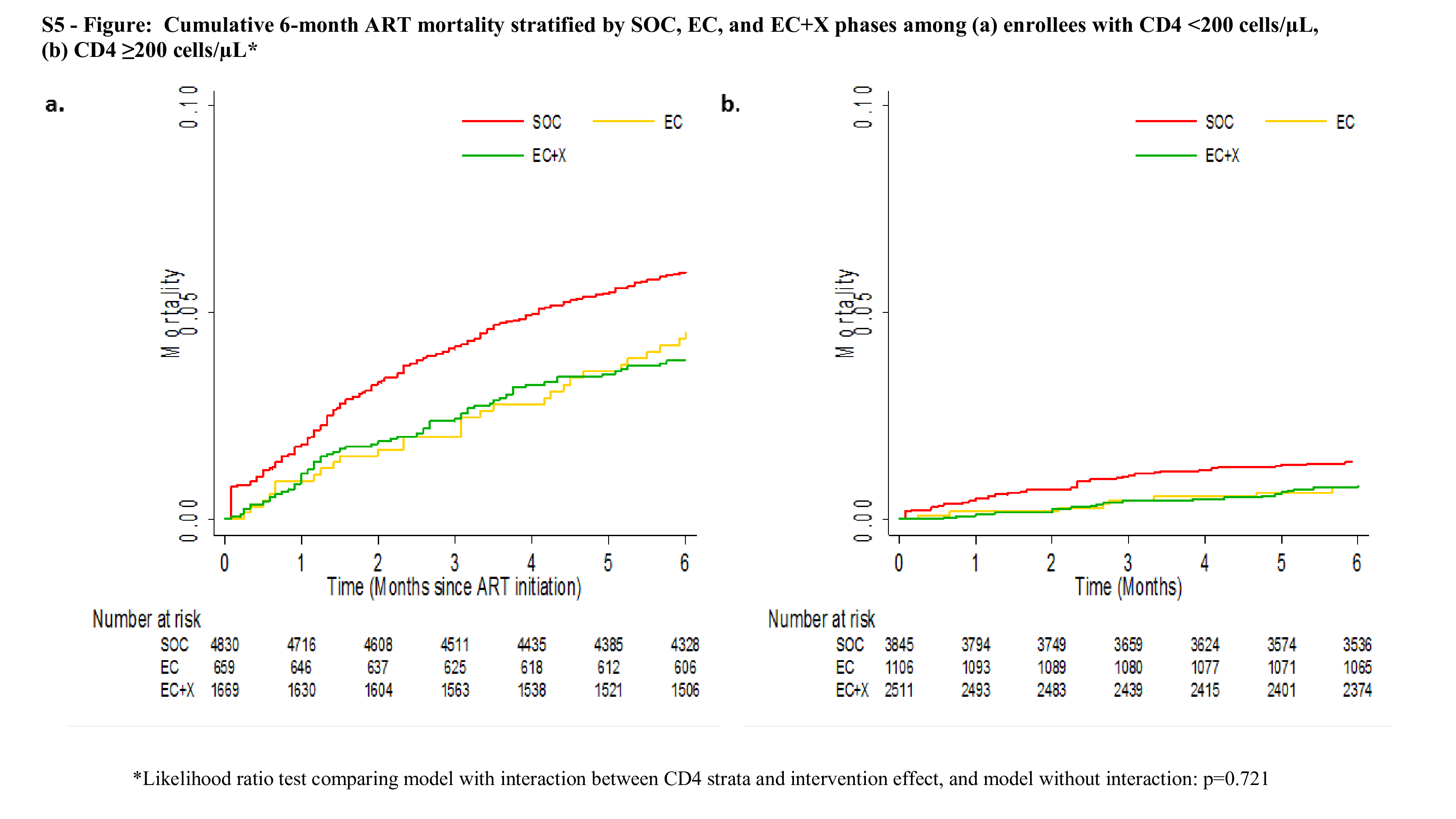

Supplement: Supplementary file 5 — Additional file 5. Figure of cumulative 6-month ART mortality stratified by SOC, EC, and EC+X phases among (a) enrollees with CD4 < 200 cells/μL, (b) CD4 ≥ 200 cells/μL. [file 12916_2019_1489_MOESM5_ESM.tiff]
