# Supplementary material for: Effect of tuberculosis screening and retention interventions on early antiretroviral therapy mortality in Botswana: a stepped-wedge cluster randomized trial
Source: BMC Med. 2020 Feb 11;18:19. doi: 10.1186/s12916-019-1489-0 (PMC7011529; doi:10.1186/s12916-019-1489-0)
Supplement: Supplementary file 6 — Additional file 6. Table of sensitivity analyses of primary and secondary study outcomes - comparison of mortality rates between study phases. [file 12916_2019_1489_MOESM6_ESM.docx]

**S6 Table: Sensitivity analyses of primary and secondary study outcomes - comparison of mortality rates between study phases**

|  |  | **Pre-specified Multivariable Model^a^** | | | | | **Model 1^b^ (Sensitivity Analysis)^a^** | | | | | **Model 2^c^ (Sensitivity Analysis)^a^** | | | | |
| --- | --- | --- | --- | --- | --- | --- | --- | --- | --- | --- | --- | --- | --- | --- | --- | --- |
|  | **Total ART enrollees** | **Total deaths incl. in adjusted analysis^d^** | **Rate/**  **100PY among patients incl. in adjusted analysis^e^** | **AHR^f^** | **(95% CI)** | **p** | **Total deaths incl. in adjusted analysis^d^** | **Rate/**  **100PY among patients incl. in adjusted analysis^e^** | **AHR^f^** | **(95% CI)** | **p** | **Total deaths incl. in adjusted analysis^d^** | **Rate/**  **100PY among patients incl. in adjusted analysis^e^** | **AHR^f^** | **(95% CI)** | **p** |
| **Primary Outcome:**  **6-month ART Mortality in SOC versus EC+X phase** | | | | | | | | | | | | | | | | |
| SOC | 8980 | 350 | 10.8 | 1.00 | -- | -- | 329 | 12.0 | 1.00 | -- | -- | 329 | 12.0 | 1.00 | -- | -- |
| EC+X | 4215 | 93 | 5.2 | 0.77 | (0.61-0.97) | 0.029 | 93 | 5.2 | 0.78 | (0.61-0.98) | 0.037 | 108 | 4.9 | 0.78 | (0.63-0.98) | 0.033 |
| **Secondary Outcomes:**  **12-month ART Mortality in SOC versus EC+X phase** | | | | | | | | | | | | | | | | |
| SOC | 8980 | 424 | 7.0 | 1.00 | -- | -- | 376 | 8.6 | 1.00 | -- | -- | 376 | 8.6 | 1.00 | -- | -- |
| EC+X | 4215 | 108 | 3.9 | 0.76 | (0.61-0.95) | 0.014 | 108 | 3.9 | 0.77 | (0.62-0.96) | 0.021 | 133 | 3.0 | 0.74 | (0.60-0.91) | 0.005 |
| **6-month ART Mortality in EC versus EC+X phase**^g^ | | | | | | | | | | | | | | | | |
| EC | 1768 | 43 | 5.5 | 1.00 | -- | -- | 28 | 7.2 | 1.00 | -- | -- | 28 | 7.2 | 1.00 | -- | -- |
| EC+X | 4215 | 93 | 5.2 | 1.13 | (0.63-2.03) | 0.690 | 93 | 5.2 | 0.90 | (0.42-1.95) | 0.793 | 108 | 5.0 | 0.79 | (0.41-1.50) | 0.472 |

Abbreviations: SOC, standard of care phase; EC, enhanced care phase; EC+X, enhanced care plus Xpert phase; PY, person-years; HR, hazard ratio; AHR, adjusted hazard ratio; CI, confidence interval; XPRES, Xpert Package Rollout Evaluation using a Stepped-Wedge design

^a^All Cox proportional hazards regression models included a random effect for clinic.

^b^Model 1 sensitivity analysis censored all follow-up time for ART enrollees at the time that the new phase began. For example, if an SOC enrollee started ART in the SOC phase, but 6- or 12-month follow-up time crossed over into the EC phase, the follow-up time for that patient was censored at the start of the EC phase for that clinic.

^c^Model 2 sensitivity analysis created a new time-dependent covariate to specify exposure to the contemporary intervention phase. Therefore, all follow-up time for ART enrollees is assigned to the phase in which the follow-up time occurred. For example, if an EC enrollee started ART in the EC phase, and 6- or 12-month follow-up time crossed over into the EC+X phase, all follow-up time that occurred in the EC+X phase for that patient was assigned to the EC+X phase rather than the EC phase.

^d^Represents the total deaths included in the adjusted analysis comparing mortality rates between phases. In unadjusted analyses among all enrollees, by 6 months after ART enrollment, there were 461 deaths among SOC enrollees, 54 deaths among EC enrollees, and 121 deaths among EC+X enrollees. By 12 months after ART enrollment, there were 551 deaths among SOC, and 137 deaths among EC+X enrollees.

^e^Represents the unadjusted mortality rates among enrollees included in the complete case analysis to generate the AHRs.

^f^Adjusted for the following characteristics at ART initiation: age, sex, pregnancy status, weight, CD4 count, hemoglobin, and ART regimen. Pre-specified, complete case, adjusted analysis and sensitivity analyses comparing SOC, EC and EC+X mortality rates included 7,184 SOC, 1,653 EC, and 3,861 EC+X enrollees.

^g^Analysis restricted to randomised stepped-wedge portion of the trial, fitting a Cox proportional hazards regression model to the data with the underlying time frame beginning August 2012 (the start of EC enrollment), and including a fixed effect for monthly changes in mortality rates during the first 6 months of ART.
