# Supplementary material for: Effect of tuberculosis screening and retention interventions on early antiretroviral therapy mortality in Botswana: a stepped-wedge cluster randomized trial
Source: BMC Med. 2020 Feb 11;18:19. doi: 10.1186/s12916-019-1489-0 (PMC7011529; doi:10.1186/s12916-019-1489-0)
Supplement: Supplementary file 7 — Additional file 7. Table of sensitivity analyses of primary and secondary study outcomes to account for non-response - comparison of mortality rates between study phases. [file 12916_2019_1489_MOESM7_ESM.docx]

**S7 Table: Sensitivity analyses of primary and secondary study outcomes to account for non-response - comparison of mortality rates between study phases**

|  |  | **Pre-specified Multivariable Model^a^** | | | | | **Sensitivity Analysis using Inverse Probability Weighting to Account for non-Response^ab^** | | | | | |
| --- | --- | --- | --- | --- | --- | --- | --- | --- | --- | --- | --- | --- |
|  | **Total ART enrollees accepting study enrollment** | **Total deaths incl. in adjusted analysis^c^** | **Rate/100PY among patients incl. in adjusted analysis^d^** | **AHR^e^** | **(95% CI)** | **p** | **Total ART enrollees at study clinics including those not enrolled in the study^f^** | **Total deaths incl. in adjusted analysis^g^** | **Rate/100PY among patients incl. in adjusted analysis^h^** | **AHR** | **(95% CI)** | **p** |
|  |  |  |  |  |  |  |  |  |  |  |  |  |
| **Primary Outcome:** | | | | | | |  |  |  |  |  |  |
| **6-month ART Mortality in SOC versus EC+X phase** | | | | | | |  |  |  |  |  |  |
| SOC | 8,980 | 350 | 10.8 | 1 | -- | -- | 8,980 | 350 | 10.8 | 1 | -- | -- |
| EC+X | 4,215 | 93 | 5.2 | 0.77 | (0.61-0.97) | 0.029 | 5,757 | 122 | 5.2 | 0.75 | (0.55-1.02) | 0.067 |
| **Secondary Outcomes:** | | | | | | |  |  |  |  |  |  |
| **12-month ART Mortality in SOC versus EC+X phase** | | | | | | |  |  |  |  |  |  |
| SOC | 8,980 | 424 | 7.0 | 1 | -- | -- | 8,980 | 424 | 7.0 | 1 | -- | -- |
| EC+X | 4,215 | 108 | 3.9 | 0.76 | (0.61-0.95) | 0.014 | 5,757 | 143 | 3.9 | 0.75 | (0.57-0.99) | 0.040 |
| **6-month ART Mortality in EC versus EC+X phase**^i^ | | | | | | |  |  |  |  |  |  |
| EC | 1,768 | 43 | 5.5 | 1 | -- | -- | 2,665 | 60 | 5.4 |  |  |  |
| EC+X | 4,215 | 93 | 5.2 | 1.13 | (0.63-2.03) | 0.690 | 5,757 | 122 | 5.2 | 1.12 | (0.50-2.50) | 0.785 |

Abbreviations: SOC, standard of care phase; EC, enhanced care phase; EC+X, enhanced care plus Xpert phase; PY, person-years; HR, hazard ratio; AHR, adjusted hazard ratio; CI, confidence interval; XPRES, Xpert Package Rollout Evaluation using a Stepped-Wedge design

^a^All Cox proportional hazards regression models included a random effect for clinic. All adjusted models were adjusted for the following characteristics at ART initiation: age, sex, pregnancy status, weight, CD4 count, hemoglobin, and ART regimen.

^b^Pre-specified sensitivity analysis adjusted for non-response using an inverse probability weighting approach. Inverse probability weights were calculated by applying separate adjusted logistic regression models to hospital versus clinic enrolling EC and EC+X patients, to predict the probability of being enrolled in the EC and EC+X phases. An adjusted logistic regression approach was used to estimate inverse probability weights to lower the likelihood of bias given the non-random enrollment approach.

^c^In unadjusted analysis, by 6 months after ART enrollment, there were 461 deaths among SOC enrollees, 54 deaths among EC enrollees, and 121 deaths among EC+X enrollees. By 12 months after ART enrollment, there were 551 deaths among SOC, and 137 deaths among EC+X enrollees.

^d^Represents the unadjusted mortality rates among enrollees included in the primary pre-specified complete case analysis to generate the AHRs.

^e^Pre-specified, complete case, adjusted analysis comparing SOC, EC and EC+X mortality rates included 7,184 SOC, 1,653 EC, and 3,861 EC+X enrollees.

^f^Column shows total ART enrollees in each study phase, regardless of whether they were enrolled in the study. The total number of ART enrollees included in the complete case AHR analysis after up-weighting includes 7,184 SOC, 2,375 EC, and 5,109 EC+X enrollees.

^g^Represents the number of deaths up-weighted to account for non-enrollment, that were included in the complete case AHR analysis.

^h^Represents the up-weighted unadjusted mortality rates among enrollees included in the complete case analysis to generate the AHRs.

^i^Analysis restricted to randomised stepped-wedge portion of the trial, fitting a Cox proportional hazards regression model to the data with the underlying time frame beginning August 2012 (the start of EC enrollment), and including a fixed effect for monthly changes in mortality rates during the first 6 months of ART.
