# Supplementary material for: Effect of tuberculosis screening and retention interventions on early antiretroviral therapy mortality in Botswana: a stepped-wedge cluster randomized trial
Source: BMC Med. 2020 Feb 11;18:19. doi: 10.1186/s12916-019-1489-0 (PMC7011529; doi:10.1186/s12916-019-1489-0)
Supplement: Supplementary file 8 — Additional file 8. Table showing predictors of being screened for at least one TB symptom in the standard of care phase of XPRES. [file 12916_2019_1489_MOESM8_ESM.docx]

**S8 - Table: Predictors of being screened for at least one TB symptom in the standard of care phase of XPRES**

|  | **Not Screened**  **(N=6,921)** | | **Screened**  **(N=1,700)** | | **OR^a^** | **95% CI** | **p-value** |
| --- | --- | --- | --- | --- | --- | --- | --- |
|  | n | % | n | % |  |  |  |
| **Age^b^** |  |  |  |  |  |  |  |
| Median, (IQR) | 6,911 | 35 (30-43) | 1,699 | 36 (31-44) | 1.06 | (1.00-1.13) | 0.056 |
| **Gender** |  |  |  |  |  |  |  |
| Female | 4,402 | 81% | 1,056 | 19% | 1.00 |  |  |
| Male | 2,519 | 80% | 644 | 20% | 1.07 | (0.91-1.25) | 0.445 |
| **If female, pregnant?**^c^ |  |  |  |  |  |  |  |
| No | 3621 | 80% | 914 | 20% | 1.00 |  |  |
| Yes | 781 | 85% | 142 | 15% | 0.72 | (0.53-0.99) | 0.041 |
| **Weight (Kg)**^d^ |  |  |  |  |  |  |  |
| <45 kg | 589 | 72% | 228 | 28% | 1.00 |  |  |
| 45-60 kg | 3,057 | 79% | 823 | 21% | 0.70 | (0.57-0.84) | <0.001 |
| >60 kg | 2,922 | 83% | 609 | 17% | 0.54 | (0.42-0.69) |  |
| **Baseline CD4**^e^ |  |  |  |  |  |  |  |
| <50 | 744 | 76% | 230 | 24% | 1.00 |  |  |
| 50-<200 | 2,879 | 80% | 730 | 20% | 0.82 | (0.69-0.97) | <0.001 |
| 200-<350 | 2,754 | 81% | 630 | 19% | 0.74 | (0.59-0.93) |  |
| 350-<500 | 184 | 79% | 49 | 21% | 0.86 | (0.55-1.35) |  |
| ≥500 | 120 | 88% | 17 | 12% | 0.46 | (0.25-0.84) |  |
| **Hemoglobin**^f^ |  |  |  |  |  |  |  |
| severe anemia | 292 | 75% | 96 | 25% | 1.00 |  |  |
| mild/moderate anemia | 3312 | 79% | 859 | 21% | 0.79 | (0.58-1.07) | 0.285 |
| no anemia | 2400 | 80% | 593 | 20% | 0.75 | (0.47-1.19) |  |

Abbreviations: OR, Odds Ratio; CI, confidence interval; IQR, inter-quartile range; XPRES, Xpert Package Rollout Evaluation using a Stepped-wedge design

^a^All logistic regression models specified a random effect for clinic. The P-value reported is that associated with the overall model’s likelihood chi-square test statistic.

^b^Odds ratio of being screened for TB associated with being 10 year’s older. Likelihood ratio test for departure from linearity (p=0.574). Age was missing for 10 (0%) of those not screened and 1 (0%) of those screened

^c^Restricted to female ART patients only

^d^Weight was missing for 353 (5%) of those not screened and 40 (2%) of those screened

^e^CD4 was missing for 240 (3%) of those not screened and 44 (3%) of those screened

^f^Hemoglobin was missing for 917 (13%) of those no screened and 152 (10%) of those screened
