# Supplementary material for: Effect of tuberculosis screening and retention interventions on early antiretroviral therapy mortality in Botswana: a stepped-wedge cluster randomized trial
Source: BMC Med. 2020 Feb 11;18:19. doi: 10.1186/s12916-019-1489-0 (PMC7011529; doi:10.1186/s12916-019-1489-0)
Supplement: Supplementary file 9 — Additional file 9. Table comparing 6-month ART outcomes before versus after efforts to ascertain accurate primary mortality outcome status among clients LTFU by study phase. [file 12916_2019_1489_MOESM9_ESM.docx]

**S9 - Table: Comparison of 6-month ART outcomes before versus after efforts to ascertain accurate primary mortality outcome status among clients LTFU by study phase**

|  | **Before Ascertainment of**  **Outcomes of Clients LTFU** | | | | | | **After Ascertainment of Outcomes of Clients LTFU** | | |
| --- | --- | --- | --- | --- | --- | --- | --- | --- | --- |
| **SOC 6 month ART Outcomes** | |  | | |  |  |  |  |  |
|  | **n** | | | | **N** | **%** | **n** | **N** | **%** |
|  |  | | | |  |  |  |  |  |
| Alive | 7,956 | | | | 8,980 | 89% | 8,125 | 8,980 | 90% |
| Dead | 322 | | | | 8,980 | 4% | 461 | 8,980 | 5% |
| LTFU | 336 | | | | 8,980 | 4% | 28 | 8,980 | 0% |
| Transfer Out | 366 | | | | 8,980 | 4% | 366 | 8,980 | 4% |
| Unable to Continue | 0 | | | | 8,980 | 0% | 0 | 8,980 | 0% |
|  |  | | | |  |  |  |  |  |
| **EC 6 month ART Outcomes** | | |  | |  |  |  |  |  |
| Alive | 1,585 | | | | 1,768 | 90% | 1,594 | 1,768 | 90% |
| Dead | 53 | | | | 1,768 | 3% | 54 | 1,768 | 3% |
| LTFU | 10 | | | | 1,768 | 1% | 0 | 1,768 | 0% |
| Transfer Out | 76 | | | | 1,768 | 4% | 76 | 1,768 | 4% |
| Unable to Continue | 44 | | | | 1,768 | 2% | 44 | 1,768 | 2% |
|  |  | | | |  |  |  |  |  |
| **EC+X 6 month ART Outcomes** | | | |  |  |  |  |  |  |
| Alive | 3,613 | | | | 4,215 | 86% | 3,641 | 4,215 | 86% |
| Dead | 119 | | | | 4,215 | 3% | 121 | 4,215 | 3% |
| LTFU | 31 | | | | 4,215 | 1% | 1 | 4,215 | 0% |
| Transfer Out | 325 | | | | 4,215 | 8% | 325 | 4,215 | 8% |
| Unable to Continue | 127 | | | | 4,215 | 3% | 127 | 4,215 | 3% |

Abbreviations: SOC, standard of care; EC, enhanced care; EC+X, enhanced care plus Xpert; LTFU, loss to follow-up (>60 days late for last scheduled appointment).
