# Supplementary material for: Effect of tuberculosis screening and retention interventions on early antiretroviral therapy mortality in Botswana: a stepped-wedge cluster randomized trial
Source: BMC Med. 2020 Feb 11;18:19. doi: 10.1186/s12916-019-1489-0 (PMC7011529; doi:10.1186/s12916-019-1489-0)
Supplement: Supplementary file 10 — Additional file 10. Table showing differences in rates of uncorrected loss to follow-up in the first 6 months of ART between SOC, EC, and EC+X phases. [file 12916_2019_1489_MOESM10_ESM.docx]

**S10 - Table: Differences in rates of uncorrected loss to follow-up in the first 6 months of ART between SOC, EC, and EC+X phases**

|  | **n** | **6-month Rate/ 100PY** | **Crude HR** | **(95% CI)** | **p** | **AHR^a^** | **(95% CI)** | **p** |
| --- | --- | --- | --- | --- | --- | --- | --- | --- |
| **Phase of enrollment** |  |  |  |  |  |  |  |  |
| Standard of Care (SOC) | 8,980 | 8.3 | 1.00 |  |  | 1.00 |  |  |
| Enhanced Care (EC) | 1,768 | 1.2 | 0.14 | (0.07-0.26) | <0.001 | 0.05 | (0.02-0.15) | <0.001 |
| Enhanced Care Plus Xpert (EC+X) | 4,215 | 1.6 | 0.21 | (0.14-0.30) | <0.001 | 0.18 | (0.12-0.27) | <0.001 |

Abbreviations: ART, antiretroviral therapy; PY, person years; HR, hazard ratio; CI, confidence interval; AHR, adjusted hazard ratio

^a^Adjusted for age, sex, pregnancy status, weight at ART initiation, CD4 count at ART initiation, hemoglobin level, and ART regimen. Adjusted analysis included 7,184 SOC, 1,653 EC, and 3,861 EC+X enrollees.
